# Modeling evolutionary transitions in social insects

**DOI:** 10.7554/eLife.12721

**Published:** 2016-01-18

**Authors:** Michael Doebeli, Ehab Abouheif

**Affiliations:** 1 Department of Zoology and Department of Mathematics, University of British Columbia, Vancouver, Canadadoebeli@zoology.ubc.ca; 2 Department of Biology, McGill University, Montreal, Canada

**Keywords:** hymenoptera, social insects, evolutionary dynamics, eusociality, mathematical model, genetics, Other

## Abstract

Mathematical models based on direct fitness calculations may be able to explain important aspects of social evolution in insects.

**Related research article** Olejarz JW, Allen B, Veller C, Nowak MA. 2015. The evolution of non-reproductive workers in insect colonies with haplodiploid genetics. *eLife* **4**:e08918. doi: 10.7554/eLife.08918

**Image** An ant queen with female workers in a colony of carpenter ants

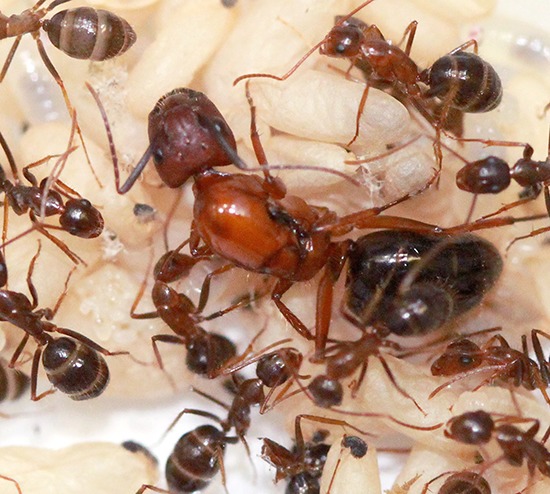

Social organization is a fundamental feature of many organisms, including humans. Eusociality is a form of social organization that has evolved in some animals, notably ants, bees and termites, and that involves, among other things, some individuals foregoing opportunities to reproduce so that they can care for the offspring of others (Hölldobler and Wilson, 1990; 2009). The existence of these ‘worker’ individuals is an evolutionary conundrum that has long fascinated biologists. How can genes that encode help to others, at the cost of not being transmitted to future generations, survive in the long run? How can genes that make their carriers sterile be a successful evolutionary invention?

Such genes can only survive in a population if they are expressed in some of the individuals that carry them, but not in others. Then these genes may be passed on if the workers preferentially help other members of the population that also carry these genes but do not express them (i.e., are not workers). The theory of kin selection introduced a concept called ‘inclusive fitness’ to explain this: inclusive fitness takes into account not only the reproductive success of a particular individual who carries a gene for helping others, but also the reproductive success of the other individuals who receive the help and carry the same gene (Hamilton, 1964).

Inclusive fitness theory predicts that the genes responsible for the forms of helping behavior displayed by worker insects can successfully spread in a population if the workers are sufficiently closely related to the individuals they help. However, in 2010, in an article that sparked fierce debate, Martin Nowak, Corina Tarnita and Edward Wilson argued that direct fitness methods – which simply calculate the average fitness of all the carriers of a gene – are better suited to explaining the evolution of eusociality than inclusive fitness theory (Nowak et al., 2010).

As a case in point, inclusive fitness theory has led to the ‘common wisdom’ that sterile workers are more likely to evolve in eusocial insects if the reproductive female that founds the colony (the queen) mates only once (a phenomenon known as monandry). Multiple matings of the queen would reduce the relatedness of individuals in the colony, and hence would reduce the inclusive fitness of the genes that cause sterility. Now, in eLife, Jason Olejarz, Benjamin Allen, Carl Veller and Nowak show that this need not be the case (Olejarz et al., 2015).

Using a population genetic model that is based on direct fitness calculations, Olejarz et al. show that the evolution of non-reproductive workers is driven by the relationship between the number of sterile workers in a colony and the number of reproductive individuals (queens and males) the colony produces. The new results show that monandry is not necessary for the evolution of non-reproductive workers. Moreover, under some conditions, the evolution of non-reproductive workers actually requires the queen to mate with multiple males. These apparently paradoxical results were obtained by considering the effects of the non-reproductive workers on the colony as a whole.

How can we apply these findings to learn more about specific clades of eusocial insects? In ants, the evolution of eusociality has occurred in a series of major transitions, each of which is thought to mark an increase in the complexity of the female castes in ant societies (Wheeler, 1986; Wilson, et al., 2005; Khila and Abouheif, 2010). The first transition gave rise to societies composed of a winged caste, consisting of the queen and male ants, and a wingless female worker caste. With the exception of wings, however, the queen and the workers are remarkably similar: the workers can mate and can lay both male and female eggs.

A second major transition, often called the ‘point of no return’, gave rise to female workers with a reduced reproductive capacity: these workers are not able to mate and can only lay unfertilized, haploid eggs that develop into males. A third major transition occurred in only a few genera of ants (out of a total of almost 300 genera) and gave rise to sterile female workers that have completely lost their ovaries. This transition is often associated with species that are ecologically and evolutionarily successful, such as fire ants (*Solenopsis invicta*) and big-headed ants (*Pheidole*).

In this context, the model of Olejarz et al. would not apply to the first transition, but it could be relevant to the second or third transitions, during which the reproductive capacity of the workers is reduced. This is of interest in light of recent work by Abderrahman Khila and one of us (EA) which identified a molecular mechanism – known as ‘reproductive constraint’ – that suppresses the production of viable male embryos in workers, but at the same time allows the production of fat-filled eggs that serve as a food source for the colony (Figure 1).

**Figure 1.**
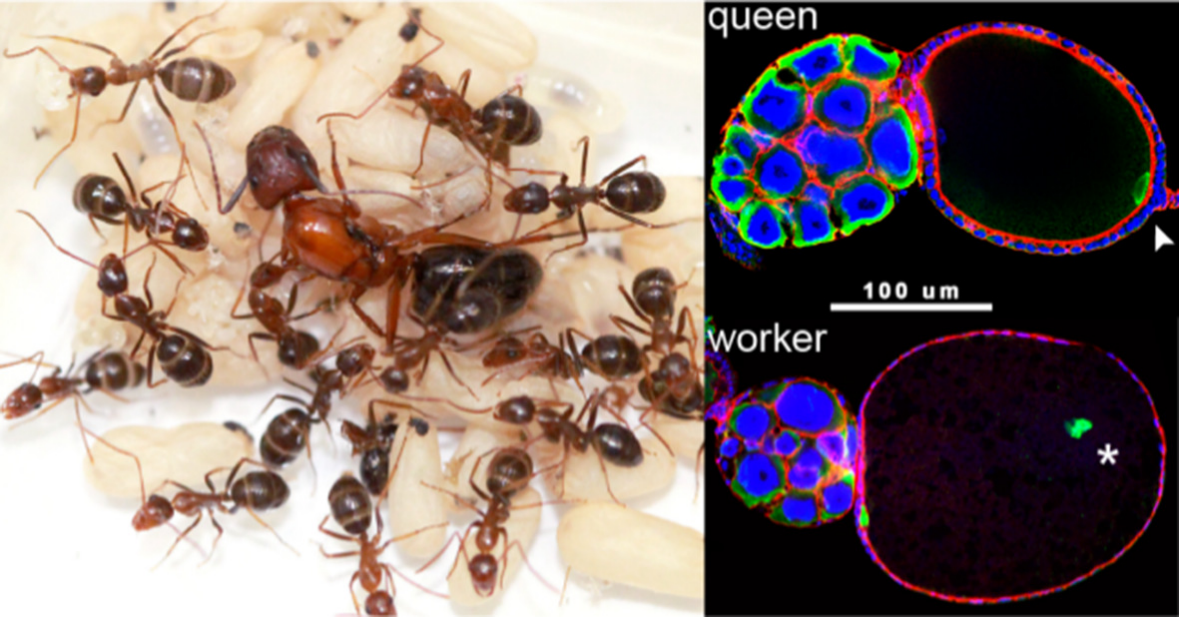
Evolving past the ‘the point of no return’. The left panel shows a colony of ants (of the species *Camponotus floridanus*) with a queen (the large individual in the center) surrounded by her female worker ants. Understanding how worker ants evolved to have a reduced reproductive capacity is a major challenge in biology. The right panel shows the position of a protein called Vasa (green) in oocytes from a normal queen (top) and a worker (bottom): *vasa* is a highly conserved developmental gene that specifies the germ cells in all animals and is necessary for fertility. In the normal queen oocyte the Vasa protein is correctly positioned at the posterior pole of the oocyte (white arrowhead). However, in many worker oocytes the Vasa protein (white star) is not in the correct position, and this leads to developmental problems, including a reduced capacity to produce viable male offspring (Khila and Abouheif, 2008). The presence of this ‘reproductive constraint’ in worker ants but not in queens allows the worker ants to use their ovaries to produce eggs that can be used to feed the colony. (Image credits: Guy L’Heureux [left]; Abderrahman Khila [right])

The model of Olejarz et al. provides a theoretical basis for understanding how reproductive constraint and other molecular mechanisms could be favored by selection, and thus could lead to major transitions in social evolution. Their results imply that selection at the colony level may be crucial for the evolution of eusociality, and point to the need for models in which both individuals and colonies are viewed as units of selection (see, for example, Simon et al., 2013).
